# Bilateral acute depigmentation of the iris (BADI) and bilateral acute iris transillumination (BAIT): A case series from a center in Brazil

**DOI:** 10.5935/0004-2749.2023-0219

**Published:** 2024-03-27

**Authors:** Flavia Veiga Costa, Amanda Gomes e Silva, Leticia Alcântara Pedroso, Ana Luiza Biancardi, André Luiz Land Curi

**Affiliations:** 1 Laboratory of Infectious Diseases in Ophthalmology, Instituto Nacional de Infectologia, Fundação Oswaldo Cruz, Rio de Janeiro, RJ, Brazil

**Keywords:** SARS-CoV-2, Pigment epithelium of eye, Iris diseases/pathology, Transillumination, Ocular hypertension, Intraocular pressure, Drug related side effect and adverse reactions, Iris diseases/drug therapy, Moxifloxacin/therapeutic Use, Humans, Case reports

## Abstract

Bilateral acute depigmentation of the iris and bilateral acute iris transillumination (BAIT) are similar clinical entities. The former causes acute-onset depigmentation of the iris stroma without transillumination, whereas the latter causes depigmentation of the iris pigment epithelium with transillumination. The etiopathogenesis of these conditions is not yet fully understood, but the proposed causes include the use of systemic antibiotics (especially moxifloxacin) and viral triggers. We present a case series of five female patients with a mean age of 41 (32-45) years, all of whom suffered acute onset of bilateral pain and redness of the eyes after moxifloxacin use (oral or topical). It is important for ophthalmologists to be aware of the two forms of iris depigmentation since this case series suggests that SARS-CoV-2 or its empirical treatment with moxifloxacin may trigger iris depigmentation. If this is the case, clinicians will likely see increased incidences of bilateral acute depigmentation of the iris and bilateral acute iris transillumination during and after the COVID-19 pandemic.

## INTRODUCTION

Bilateral acute depigmentation of the iris (BADI) and bilateral acute iris transillumination (BAIT) share certain features. BADI is characterized by acute-onset depigmentation of the iris stroma without transillumination^(1)^, while BAIT manifests as depigmentation of the iris pigment epithelium with transillumination. In both conditions, the pigment deposits released into the trabecular meshwork often result in increased intraocular pressure (IOP), although this occurs more often in BAIT^(2)^.

The etiology of these two syndromes is yet to be fully elucidated. However, previous research has found their onset to be associated with the use of antibiotics, such as systemic quinolones, especially moxifloxacin. Others have posited viral infections as a possible trigger^(3)^. We describe five cases of BAIT and BADI, four of which occurred after treatment with oral moxifloxacin and one after topical moxifloxacin.

## CASE REPORTS

We searched the medical records of a tertiary uveitis center in Brazil (Evandro Chagas National Institute of Infectious Diseases - INI/ Fiocruz) for patients who had received a diagnosis of BAIT or BADI between June 2014 and May 2022. The records of five patients were extracted, and detailed ocular data and medical histories were obtained for each patient. At each visit to the center, patients underwent complete ocular examinations. These included best-corrected visual acuity (BCVA), slit-lamp biomicroscopy, tonometry, and fundoscopy. The corneal sensation was evaluated using cotton yarn. This study was performed following the tenets of the 2013 revision of the Declaration of Helsinki and approved by the ethics committee of our institution. The informed consent requirement was waived by the committee owing to the retrospective, noninterventional nature of the study.

## RESULTS

All five patients in this series were women with a mean age of 41 years (range, 32-45) years. In each patient, both eyes were simultaneously involved or within a few days of one another. All patients had an unremarkable ocular history. Four of the patients had recently used oral moxifloxacin, and one had used topical moxifloxacin.

In all of the patients, pigmented cells were observed in the anterior chamber. The bilateral BCVA was 1.0 (20/20) in four cases and 20/25 in one.

Three patients had bilateral elevation of their IOP (≥21 mmHg) on their first visit. One of the other two was close to the upper limit 20/18 mmHg), and the other (18/18 mmHg) had already begun treatment with triple topical antiglaucoma medication (timolol, brimonidine, and dorzolamide).

No inflammatory cells were found in the anterior chamber or synechiae of any of the patients during any of their visits to the center. Corneal sensation was intact, and the posterior segment was normal in all. Transillumination was found in three patients, leading to the diagnosis of BAIT, the remaining two were diagnosed with BADI.

In those using steroids, the dosages were slowly tapered. Hypotensive agents were prescribed to those with an IOP above the normal limit. Unfortunately, patient 3 experienced changes in her visual field, typical of glaucoma, so her brimonidine treatment was continued. Despite drug treatment, patient 5 required a tube-shunt implante.

**Figure 1:**
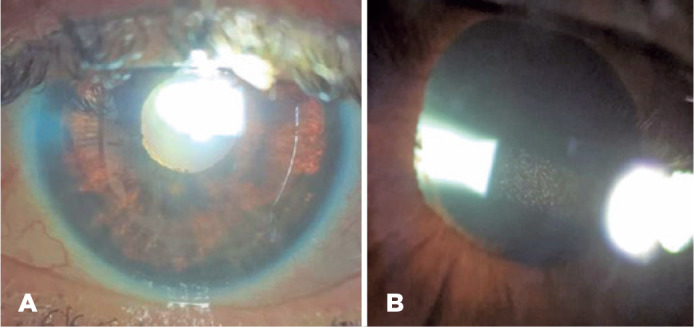
(A) Transillumination; (B) Pigmented cells were observed circulating in the anterior chamber

The demographic and medical characteristics of the five cases are summarized in table 1. Figure 1 depicts the images from patient 4.

**Table 1:** Demographic and clinical characteristics of the patients in this study

	Case 1	Case 2	Case 3	Case 4	Case 5
Sex	Female	Female	Female	Female	Female
Age	45y	32y	40y	45y	43y
ATB	oral moxifloxacin	oral moxifloxacin	oral moxifloxacin	oral moxifloxacin	topical moxifloxacin
Reason ATB	COVID-19	pneumonia	sinus infection	COVID-19	Conjunctivitis
Red eye	yes	yes	yes	yes	yes
Photophobia	no	yes	yes	no	yes
Ocular pain	yes	yes	yes	yes	yes
Blurred vision	no	no	no	yes	yes
Circulating pigment in the AC	yes	yes	yes	yes	yes
Transillumination	yes	no	no	yes	yes
Additional findings	Pupillary distortion	Diffuse depigmentation	Diffuse depigmentation	Pupillary distortion	Pupillary distortion
Initial BCVA right	20/20	20/20	20/25	20/20	20/20
Initial BCVA left	20/20	20/20	20/25	20/20	20/20
Final BCVA right	20/20	20/20	20/20	20/20	20/20
Final BCVA left	20/20	20/20	20/20	20/20	20/20
IOP (mmHg)	48 (OD)/ 50 (OS)	20 (OD)/ 18 (OS)	55 (OD)/ 55 (OS)	18^*^(OD)/ 18^*^(OS)	37 (OD)/ 35 (OS)
Corneal sensation intact	yes	yes	yes	yes	yes
Laterality	bilateral	bilateral	bilateral	bilateral	bilateral

## DISCUSSION

The five patients in the present study were females in their forties who presented with an acute onset of red, painful eyes after the use of moxifloxacin. These findings were consistent with those of Tugal-Tutkun and Urgancioglu, who published a case series in 2006 describing the first five cases of BADI^(1)^. In 2011, Tugal-Tutkun et al. published a further 26 cases with a similar presentation but with additional iris transillumination, this was named BAIT^(2)^.

Despite their similarities, the typical characteristics of BADI and BAIT allow differentiation between them. In BADI, there is no iris transillumination as there is no epithelial involvement, just a diffuse or geographic depigmentation of the iris stroma, while BAIT presents as transillumination defects and pupillary abnormalities^(3^,^4)^.

BADI is known to have a better prognosis and a lower incidence of ocular hypertension^(4)^. However, in the present study, the highest level of IOP was observed in a BADI case. Nonetheless, increased IOP occurs earlier in BAIT and tends to be refractory to treatment, sometimes requiring filtering surgery. To lower the incidence of complications, all BAIT and BADI patients should be kept under regular observation for IOP during and a little after the complete resolution of pigment circulation, which may persist for 1-18 months (median, 5 months)^(4)^.

The etiology of the two syndromes remains uncertain^(3)^. The evidence for oral fluoroquinolone antibiotics, especially moxifloxacin, as causal agents suggests that they constitute a possible cause only. While the adverse event generally occurs within a reasonable time after the administration of the drug, it could also be plausibly explained by other drugs or chemicals, an underlying disease, or a concurrent disease^(5)^.

However, numerous cases have been reported after intracameral moxifloxacin injection^(6)^. The use of intracameral moxifloxacin for endophthalmitis prophylaxis after intraocular surgery is becoming a widely accepted practice^(7)^. Therefore, it is important to recognize BAIT and BADI as possible consequences or complications, particularly in procedures where the native lens is left intact. It has been hypothesized that phakic patients are at a higher risk of developing BAIT or BADI, most likely because either posterior-to-anterior clearance is impaired or because posterior synechiae trap the drug in the posterior chamber, resulting in prolonged exposure of the iris pigment epithelium^(8)^.

A case of BAIT has been reported after the incorrect use of topical moxifloxacin. The patient had presented with an ophthalmological emergency 1 month prior with purulent discharge, itching, and conjunctival hyperemia. She was diagnosed with bacterial conjunctivitis and prescribed hourly doses of topical moxifloxacin for 10 days. She reported a partial improvement in her symptoms but also experienced new symptoms, including ocular pain and photophobia. The patient continued to selfadminister moxifloxacin hourly until she was able to obtain further medical advice. Her medical history suggested an overlap between bacterial conjunctivitis and BAIT.

Moxifloxacin is one of the most frequently administered topical antibiotics after cataract surgery^(9)^ and appears to be safe at the correct dosage.

It is important to note that two of our patients developed BAIT after the SARS-CoV-2 infection. There have been previous reports linking these two entities^(3)^. However, the present study cannot corroborate this relationship since both patients used prescription oral moxifloxacin as an empirical antiviral against both DNA and RNA viruses^(10)^. This casts doubt on whether the cause of BAIT was moxifloxacin therapy, viral trigger, or both.

Both BAIT and BADI must be differentiated from iridocyclitis and other diseases that cause pigment dispersion in the anterior chamber. It is vital to make a correct differential diagnosis of BAIT and BADI from anterior uveitis to avoid the unnecessary use of corticosteroids and minimize the risk of IOP elevation.

Ophthalmologists must be cognizant of the fact that moxifloxacin use is likely to have increased during the pandemic, which is likely to provoke a corresponding increase in the incidence of BAIT and BADI.
